# Deep learning-based improved side-channel attacks using data denoising and feature fusion

**DOI:** 10.1371/journal.pone.0315340

**Published:** 2025-04-09

**Authors:** Hai Huang, Jinming Wu, Xinling Tang, Shilei Zhao, Zhiwei Liu, Bin Yu

**Affiliations:** 1 School of Computer Science and Technology, Harbin University of Science and Technology, Harbin, China; 2 Collaborative Innovation Center Laboratory for Artificial Intelligence Application Technology, Harbin, Heilongjiang, China; Mae Fah Luang University, THAILAND

## Abstract

Deep learning, as a high-performance data analysis method, has demonstrated superior efficiency and accuracy in side-channel attacks compared to traditional methods. However, many existing models enhance accuracy by stacking network layers, leading to increased algorithmic and computational complexity, overfitting, low training efficiency, and limited feature extraction capabilities. Moreover, deep learning methods rely on data correlation, and the presence of noise tends to reduce this correlation, increasing the difficulty of attacks. To address these challenges, this paper proposes the application of an InceptionNet-based network structure for side-channel attacks. This network utilizes fewer training parameters. achieves faster convergence and demonstrates improved attack efficiency through parallel processing of input data. Additionally, a LU-Net-based network structure is proposed for denoising side-channel datasets. This network captures the characteristics of input signals through an encoder, reconstructs denoised signals using a decoder, and utilizes LSTM layers and skip connections to preserve the temporal coherence and spatial details of the signals, thereby achi-eving the purpose of denoising. Experimental evaluations were conducted on the ASCAD dataset and the DPA Contest v4 dataset for comparative studies. The results indicate that the deep learning attack model proposed in this paper effectively enhances side-channel attack performance. On the ASCAD dataset, the recovery of keys requires only 30 traces, and on the DPA Contest v4 dataset, only 1 trace is needed for key recovery. Furthermore, the proposed deep learning denoising model significantly reduces the impact of noise on side-channel attack performance, thereby improving efficiency.

## Introduction

Side channel attackers exploit leaked information related to keys in the physical implementation of encryption algorithms, such as sound, visible light, electromagnetic radiation, and timing information, to recover the system’s keys [1]. Generally, side-channel attacks can be classified into two types: non-profiled attacks and profiled attacks. The former involves analyzing leaked information directly from the target device to extract key-related data, while the latter necessitates acquiring a duplicate of the target device to discern key-related features before initiating an attack on the original device. Given deep learning’s sensitivity to data features, the utilization of deep learning algorithms can significantly improve the efficiency and precision of side-channel attacks. The minimum number of power traces needed to successfully recover the key and the amount of training time required for the model to operate at its best are commonly used metrics to assess attack efficiency.

In 2016, Maghrebi et al. [2] demonstrated that deep learning techniques for analyzing power consumption traces outperformed template attacks, machine learning models, and long short-term memory network models. In 2020, Benadjila et al. [3] conducted an in-depth study on the hyperparameter selection of models using deep learning in side-channel attack scenarios. They proposed a convolutional neural network model CNN_best based on the VGG-16 network architecture for image recognition. The CNN_best model exhibited good feature extraction and classification capabilities on the ASCAD dataset after structural and parameter adjustments. In the same year, Yang et al. [4] optimized the CNN_best model by introducing methods such as batch normalization and dropout, enhancing the model’s generalization in side-channel attacks. In 2021, Zaid et al. [5] introduced an efficient method for constructing CNN architectures, achieving improved efficiency. The model proposed in their study recovered the key with 170 power traces on the ASCAD dataset and only 3 power traces on the DPA contest v4 dataset. In 2022, Zheng et al. [6] proposed a new convolutional neural network structure, CBAPD, based on the characteristics of the target attack data. The CBAPD model was successfully attacked with 50 power traces on the ASCAD dataset and 3 power traces on the DPA contest v4 dataset.

The above-mentioned paper employs convolutional neural networks (CNNs) as the foundation, using a simple stacking of network layers to enhance image recognition accuracy. However, during the training phase, these CNNs often overly focus on non-critical areas around informative regions, which lack utility and may even interfere with the model’s prediction results, affecting the accuracy improvement of the model. This paper proposes a novel network structure based on the improved Inception_v3 model. The network utilizes multiple convolutional kernels of different sizes, performing convolution and pooling operations in parallel on input data. Finally, all results are concatenated, allowing the model to simultaneously focus on features at different scales for better data representation [7]. The proposed model was tested on the ASCAD synchronous dataset and the DPA Contest v4 dataset, effectively improving side-channel attack efficiency.

While the proposed InceptionNet model achieves good results in attacking noise-free side-channel datasets, deep learning attack methods pose serious threats to the security of encryption devices but still have significant limitations. These attack methods rely on the correlation features of signals, and a decrease in correlation severely impacts their effectiveness. Therefore, a series of countermeasures and protection methods against side-channel attacks continue to be proposed. These include introducing random noise, employing physical shielding techniques, etc., to reduce signal correlation and increase the difficulty of side-channel attacks.

Reducing noise interference is a key focus of data preprocessing in side-channel attacks. In 2010, Souissi et al. [8] proposed a new technique based on Kalman theory and demonstrated that the efficiency of power analysis attacks can be improved by Kalman filtering. In 2013, Feng et al. [9] proposed an emd-based denoising method and investigated its effectiveness in filtering out high-frequency noise targeting side-channel attacks. In 2014, Liu et al. [10] proposed a noise reduction method based on wavelet analysis to improve the performance of correlation power analysis, and their results showed that the wavelet transform has a better noise reduction effect compared to the higher-order cumulants. In 2016, Yao et al. [11] proposed a signal feature extraction met-hod based on the combination of empirical mode decomposition (EMD) and the singular value difference spectrum. The feature information of the signal can be extracted, and the signal-to-noise ratio of the signal can be effectively improved. In 2020, Duan et al. [12] applied the translation invariant wavelet method based on wavelet analysis with the wavelet mode maxima method to the preprocessing of power consumption curves, which improved the efficiency of power consumption analysis. In 2023, Zhao et al. [13] proposed a denoising method using K-Singular Value Decomposition Dictionary Learning. Through multiple iterations, a set of basic elements that best represent the signal features was found. These elements were then used to reconstruct the signal, thereby removing the influence of noise. The method has been proven to exhibit greater flexibility and adaptability to various signal types and noise compared to wavelet denoising. Wang et al. [14] proposed a hybrid threshold denoising framework based on singular value decomposition for data preprocessing for side channel analysis. The signal-to-noise ratio of traces is effectively improved.

Although these techniques are useful, they often require prior knowledge of noise characteristics to better distinguish signals from noise and achieve denoising purposes. Additionally, the differences in signal and noise characteristics across different devices make this problem extremely complex. In 2020, Wu et al. [15] introduced a new method that utilizes denoising autoencoders (DAE) to eliminate noise. This marked the first use of deep learning to denoise side-channel datasets, demonstrating significant advantages in white-box scenarios. However, the effectiveness of attacks after denoising was notably reduced compared to the pre-noise addition. To address this, this paper proposes a novel network structure, LU-Net, applied to denoise side-channel datasets. This network model adopts an encoder-decoder structure, where the encoder captures the features of the input signal and the decoder reconstructs the denoised signal. It incorporates LSTM layers and skips connections to maintain the temporal coherence and spatial details of the signal, achieving the goal of denoising. Post-denoising, the absence of noise allows for improved efficiency in side-channel attacks.

The contributions of this paper can be summarized as follows:

- We introduce how deep learning can be applied to side-channel attacks and preprocess side-channel attack datasets for denoising, making it easier for attackers to infer sensitive information.
- We propose an improved network structure based on the Inception_v3 model for side-channel attacks, demonstrating the efficiency of our algorithm on the ASCAD dataset and DPA-contest v4 dataset. In terms of trace quantity, it achieves higher efficiency compared to state-of-the-art profiled attacks.
- We introduce the LU-Net model for denoising side-channel datasets, demonstrating its effectiveness on the ASCAD dataset with the addition of different types of noise.

The structure of the remaining sections is as follows: Section 2 introduces background knowledge; Section 3 discusses the application of the proposed InceptionNet model in side-channel attacks; Section 4 presents the denoising model for side-channel datasets based on the LU-Net proposed in this paper; Section 5 covers experimental design and result analysis; and Section 6 provides the conclusion of this research.

## Background

### AES algorithm

**Fig 1:**
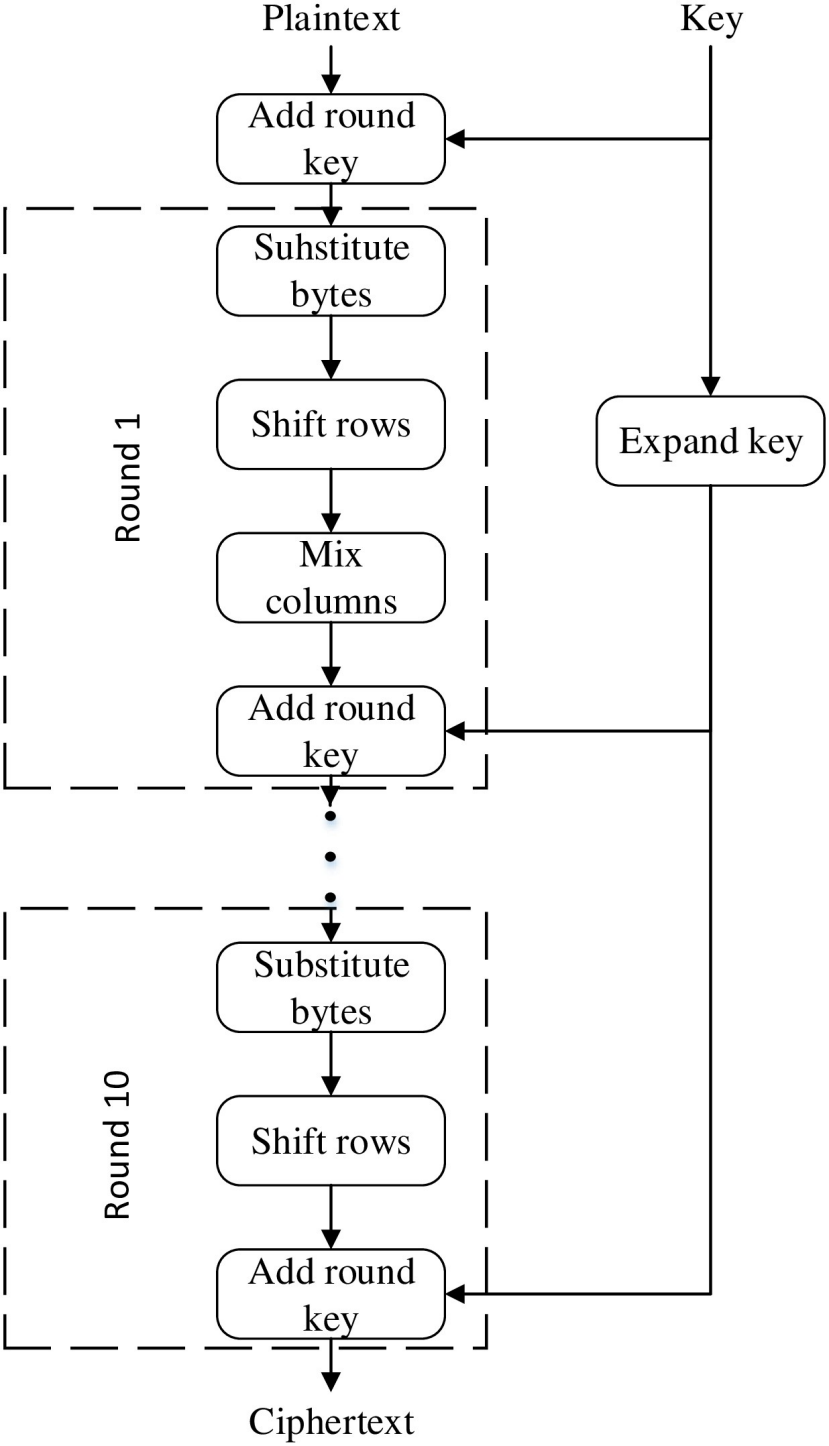
AES-128 structure.

The Advanced Encryption Standard (AES) is a block cipher algorithm. It has a block size of 128 bits, and three key lengths are available: 128 bits, 192 bits, and 256 bits. The corresponding numbers of rounds for encryption are 10 rounds, 12 rounds, and 14 rounds, respectively. The round structure involves four basic transformations: AddRoundKey, ShiftRows, MixColumns, and SubBytes [16]. Taking the example of the 128-bit AES encryption algorithm, the computational process is shown in Fig 1.

Row shifting involves circular shifting operations on different rows of the intermediate state in the AES encryption algorithm. The detailed computational process is illustrated in Formula 1 below. The shifting pattern follows the rule of performing a left shift of 0 bytes for the first row, a left shift of 1 byte for the second row, a left shift of 2 bytes for the third row, and a left shift of 3 bytes for the fourth row.

Column mixing involves multiplying the matrix resulting from the second step of the transformation by a constant matrix. Each byte in every column is mapped to a new value through this operation, and the multiplication is performed within the finite field GF(2^8^) [17]. The specific computation is illustrated in Eq (2):

$$\begin{array}{c} \left[\begin{array}{cccc} X_{0} & X_{1} & X_{2} & X_{3}\\ X_{4} & X_{5} & X_{6} & X_{7}\\ X_{8} & X_{9} & X_{10} & X_{11}\\ X_{12} & X_{13} & X_{14} & X_{15} \end{array}]\to [\begin{array}{cccc} X_{0} & X_{1} & X_{2} & X_{3}\\ X_{5} & X_{6} & X_{7} & X_{4}\\ X_{10} & X_{11} & X_{8} & X_{9}\\ X_{15} & X_{12} & X_{13} & X_{14} \end{array}\right] \end{array}$$ (1)

$$\begin{array}{c} \left[\begin{array}{cccc} S_{00}^{\prime } & S_{01}^{\prime } & S_{02}^{\prime } & S_{03}^{\prime }\\ S_{10}^{\prime } & S_{11}^{\prime } & S_{12}^{\prime } & S_{13}^{\prime }\\ S_{20}^{\prime } & S_{21}^{\prime } & S_{22}^{\prime } & S_{23}^{\prime }\\ S_{30}^{\prime } & S_{31}^{\prime } & S_{32}^{\prime } & S_{33}^{\prime } \end{array}]=[\begin{array}{cccc} 02 & 03 & 01 & 01\\ 01 & 02 & 03 & 01\\ 01 & 01 & 02 & 03\\ 03 & 01 & 01 & 02 \end{array}][\begin{array}{cccc} S_{00} & S_{01} & S_{02} & S_{03}\\ S_{10} & S_{11} & S_{12} & S_{13}\\ S_{20} & S_{21} & S_{22} & S_{23}\\ S_{30} & S_{31} & S_{32} & S_{33} \end{array}\right] \end{array}$$ (2)

Round key addition involves performing a bitwise XOR operation between the 16-byte round key generated after key expansion and a 4x4 byte matrix. Additionally, each round’s round key is only dependent on the original key and is independent of the round keys in other rounds.

### Deep learning side-channel attacks

The application of deep learning in side-channel attacks can be divided into two stages: the analysis stage and the attack stage. In the analysis stage, a neural network model is established and trained to explore the relationship between power traces and labels [18]. Since the collected leakage information is often noisy, misalignment of power traces or noise in the collected power traces can reduce the correlation between power traces and device processing data. Therefore, preprocessing operations on this leakage information are usually required before training, such as noise reduction, data alignment, and dimensionality reduction.

**Fig 2:**
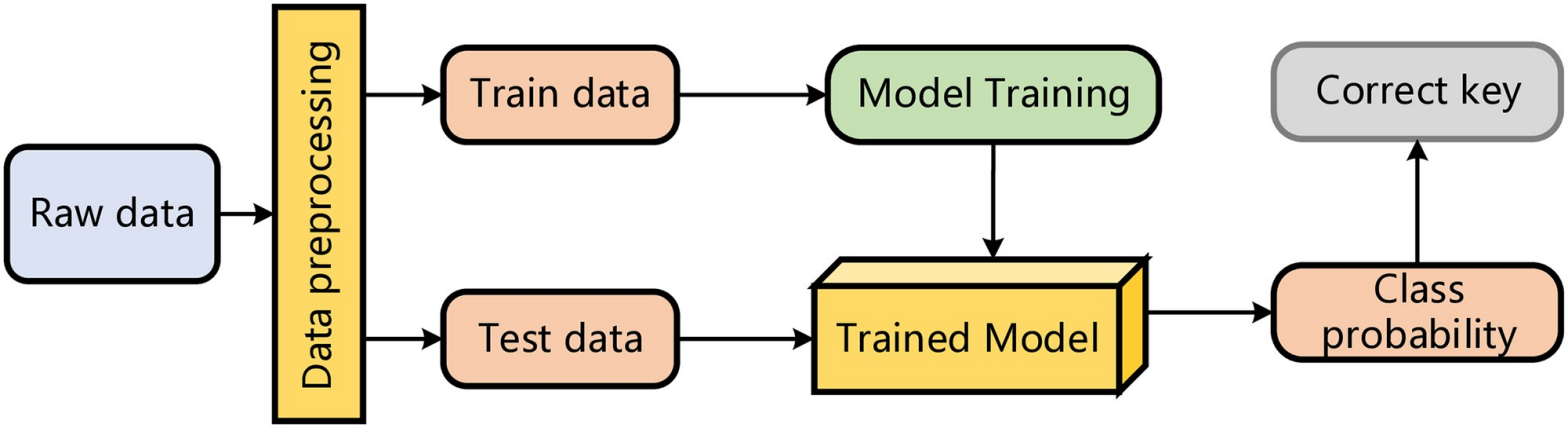
Profiling side-channel attack.

In the attack phase, the attacker uses the trained weights to determine the probability of each category, which is given by the intermediate values corresponding to each key guess. The key guess with the highest probability value indicates the most lik-ely secret key. The side-channel analysis metric Rank is used to evaluate the test results, as discussed in the next section. Typically, deep learning models take side-channel data as input, output posterior probabilities for each possible key, and then find the rank value for the correct key guess through sorting, completing the key guessing process. The process of side-channel attacks is shown in Fig 2.

Attackers leverage side-channel attack techniques to train models for analyzing information leakage from encrypted devices, thereby reconstructing key information [19]. The application of deep learning in side-channel attacks is characterized by efficiency, precision, adaptability, and other features [20]. It can be used to attack various types of encryption algorithms, leading to widespread attention in the field of information security.

### Sections and subsections

In side-channel attacks, the rank metric is a measure of the attacker’s effectiveness in guessing the correct key, which indicates the ranking of the gues-sed correct key among all possible keys and provides a more intuitive evaluation of the results. In this paper, we use the rank metric to evaluate the effectiveness of the network model proposed in this experiment in the side-channel attack. It is defined as follows:

$$\begin{array}{c} rank\left(real\_key\right)\;=\left\{i|sort(d_{k})[i]=d_{k}[real\_key]\right\} \end{array}$$ (3)

where *i*, *k* ∈ {0, 1, ⋯, 255}, and the output of the model *d*_*k*_ (the posterior probability of key k) is a tensor with 256 elements corresponding to the probability of guessing the key {0, 1, ⋯, 255}. *sort*() is used to sort *d*_*k*_ in descending order, and the index corresponding to the probability of the correct key is the rank of that key [5]. The correct key has the largest output probability, i.e., *d*_*k*_[*real*_*key*] should have the largest probability in the case of a correct attack. The correct key should be ranked first among all guessed keys, so its corresponding index should be 0. Researchers typically design a model to minimize the rank of the correct keys; the model needs to use fewer power traces to determine the correct key. The lower the number of power traces used, the more effective the attack will be.

## InceptionNet model

**Fig 3:**
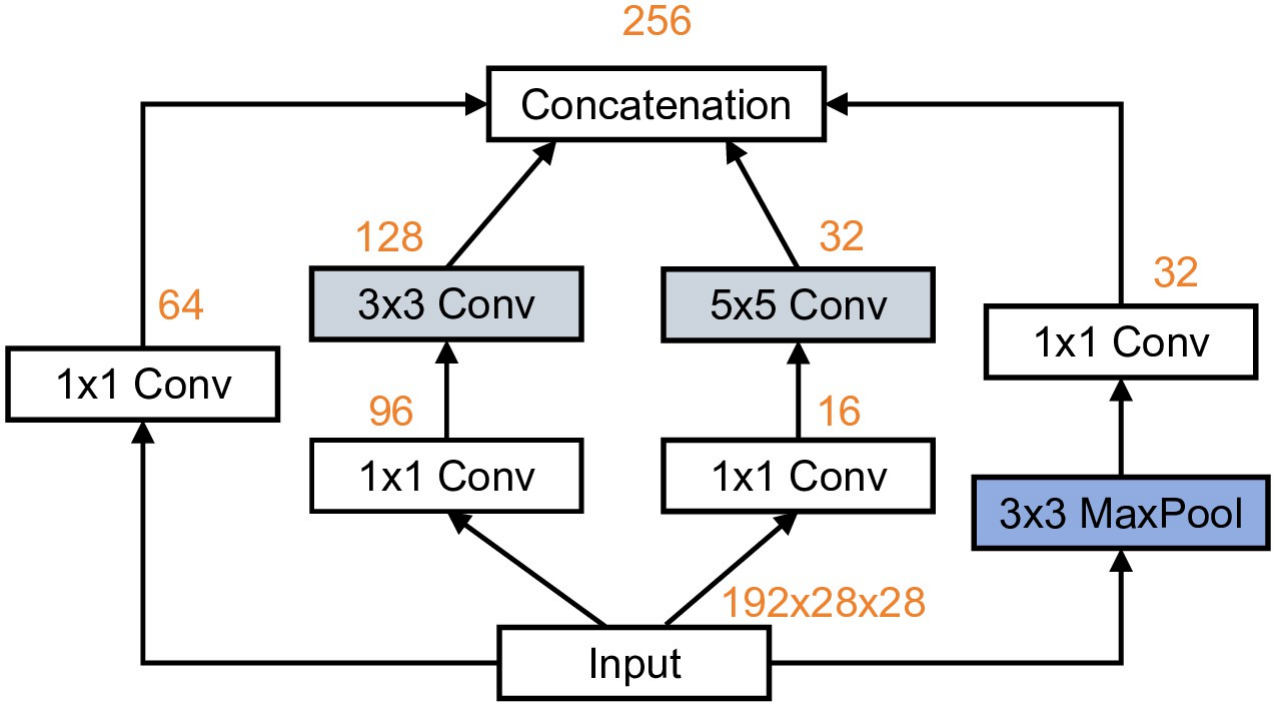
Inception block parameter change process.

InceptionNet Model Inception is a deep learning network based on the CNN architecture, which consists of convolutional layers, pooling layers, fully connected layers, and an output layer. The introduction of Inception primarily addresses the issue of excessive parameters when stacking multiple convolutional layers. As depicted in Fig 3, for an input with 192 channels and an output with 256 channels, the number of parameters for a single 3x3 or 5x5 convolutional layer is 0.44M and 1.22M, respectively, whereas the Inception block controls model complexity by reducing the number of channels, resulting in only 0.16M parameters.

**Fig 4:**
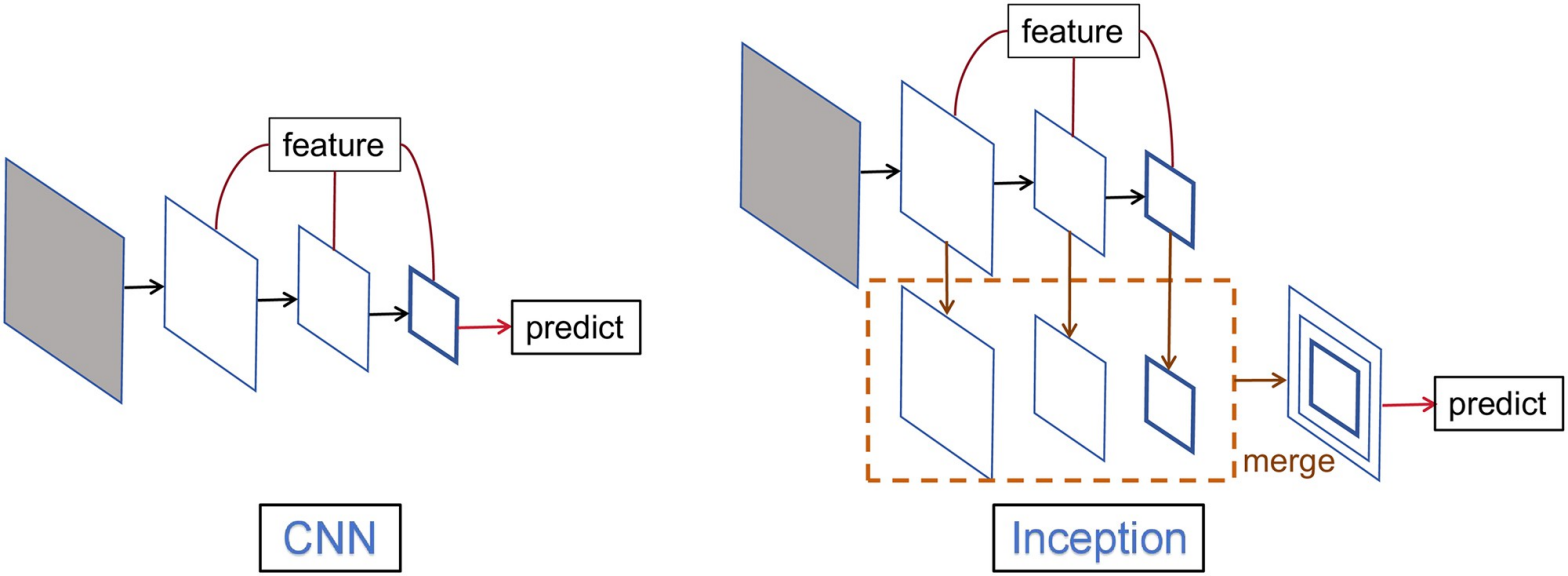
Process of feature fusion.

Inception Block Fig 4 illustrates the difference between CNN and Inception modules in the feature fusion process. The feature fusion network of Inception integrates different features extracted from deep and shallow layers using receptive fields of different scales and finally predicts using the fused features. Traditional CNNs fail to utilize features of different scales effectively. When using features from shallow layers for prediction, due to the lack of high-level semantic features, the network’s classification accuracy for small targets is lower, while predictions from deep layers lose detailed information. Features extracted at different scales have their own advantages, and we can exploit these features to accomplish various high-demand tasks.

**Fig 5:**
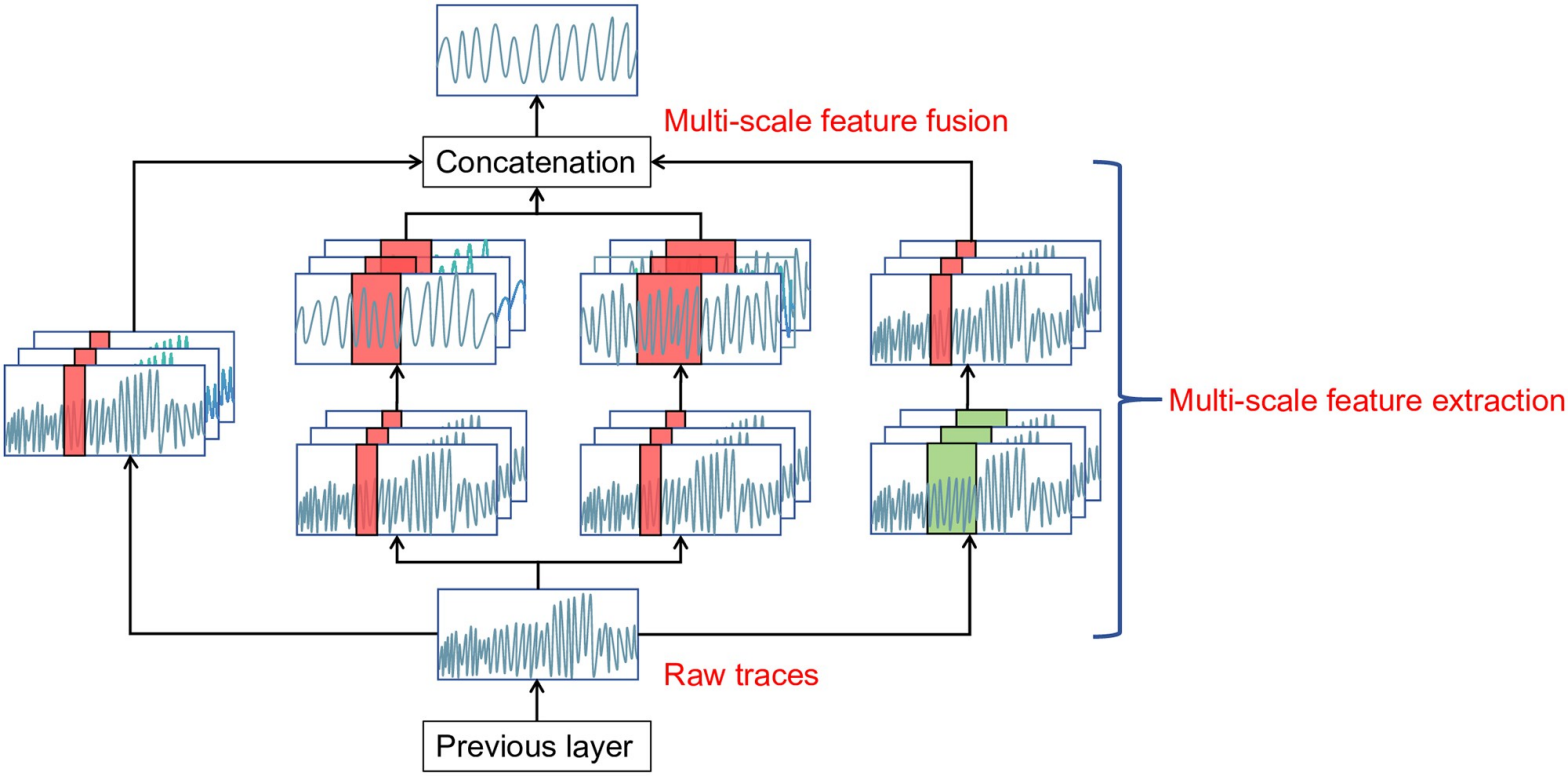
A single Inception module structures the feature extraction process.

The Inception module connects different convolutional and pooling layers in parallel and can have multiple channels within the same convolutional layer, each capable of containing convolutional kernels of different sizes and depths, enabling the network to extract features at different levels [21]. Inception networks are typically composed of several concatenated Inception modules. Fig 5 depicts the feature extraction process of a single Inception module. It uses three filters of different sizes (1x1, 3x1, and 5x1) to perform convolution operations on the input and also performs max-pooling. The outputs of all sub-layers are then concatenated and sent to the next Inception module.

**Fig 6:**
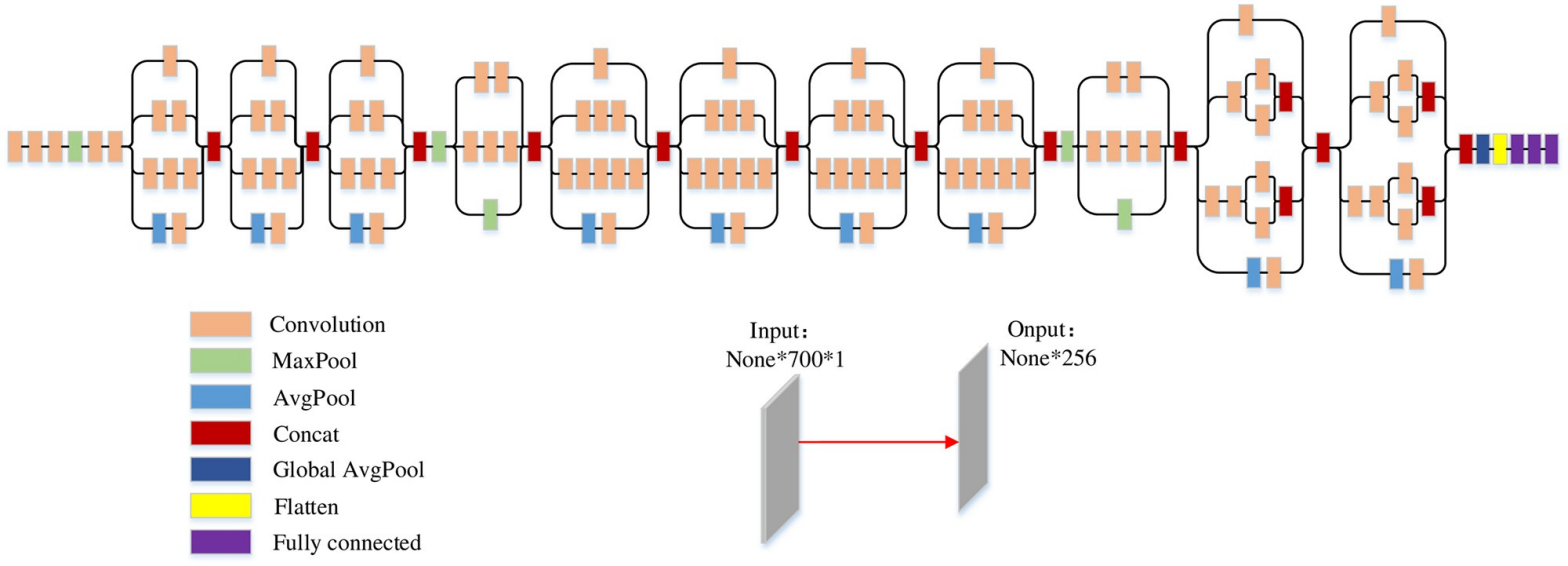
InceptionNet model.

Side-channel attack data consists of one-dimensional temporal signals. Convolution acts as a granularity non-linear augmented time-series feature extractor, perceiving various features in the power consumption at different scales through different filters, then synthesizing local information at higher levels, acquiring global information after extracting information at different scales. Pooling layers are used for downsampling to retain significant features and reduce feature dimensions. The extracted feature vectors at different scales are combined through padding operations to enhance interaction of information, improve the network’s representation capability for nonlinear features, and further learn internal correlated features of power consumption over time. Features of convolutional layers are provided throu-gh weight sharing and sparse connections, where they compute the results of neurons associated with the current local region of the previous layer. Additionally, it shares weights of neurons across different feature maps, corresponding to kernels within the same layer. In the classification part, the role of fully connected layers is to accept network feature outputs and custom-generated segmented features. The overall network structure is depicted in Fig 6.

To improve the effectiveness of the network model for side-channel attack, this paper adjusts the hyperparameters by grid traversal and selects the hyperparameters with the best performance as the final hyperparameters. The hyperparameters are shown in Table 1.

**Table 1:** Hyperparameter selection.

Hyperparameter	Value
Batch size	200
Epochs	50
Learning rate	0.00001
Leakage model	ID
Target dataset	ASCAD / DPA contest v4
Num of class	256
Num of features	256
Optimizer	RMSprop
GPU	Nvidia a40 / 48G

## LU-Net model

**Fig 7:**
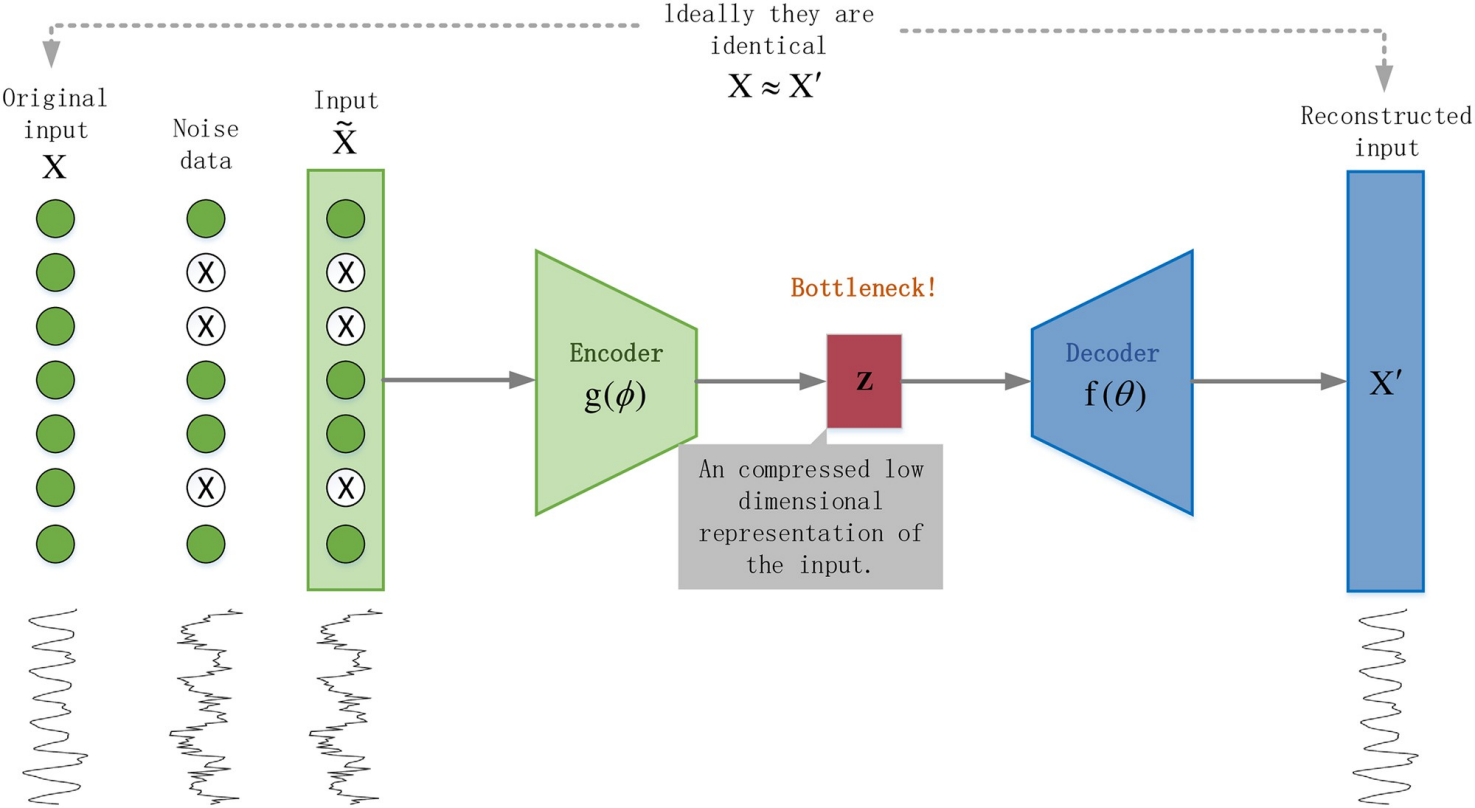
The basic architecture of an autoencoder.

By employing deep learning techniques to denoise side-channel datasets, the traces processed in this manner are more conducive to side-channel attacks. The autoencoder, a classic unsupervised deep learning model, aims to learn abstract features of input samples by maintaining consistency between the network’s output and input samples. Fig 7 illustrates the fundamental structure of an autoencoder, which is typically composed of an encoder and a decoder. The encoder maps high-dimensional input samples to a low-dimensional abstract representation, achieving sample compression and dimensionality reduction. Meanwhile, the decoder transforms this abstract representation back to the expected output, thereby reproducing the input samples [22]. The bottleneck represents the lowest possible dimensionality of the input data, serving to restrict the flow of information from the encoder to the decoder, allowing only the most critical information to pass through. During this process, structural details may be lost, but the most essential parts are retained.

**Fig 8:**
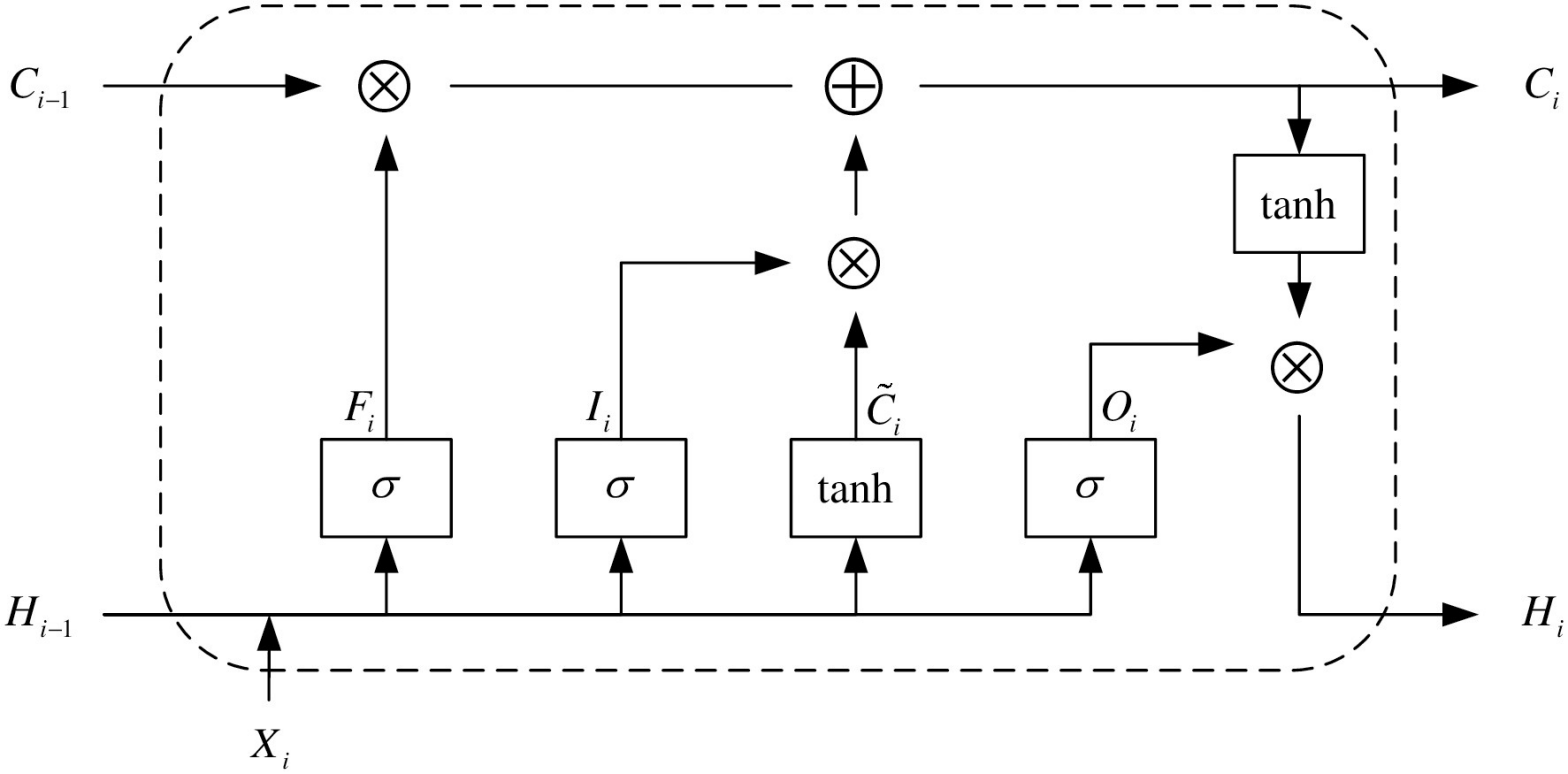
Long short-term memory network structure diagram.

The leaked information in side-channel attacks typically manifests as one-dimensional time-series signals, which exhibit long-term dependencies, meaning past information significantly influences future predictions. Long Short-Term Memory (LSTM) networks, equipped with built-in memory units, are capable of capturing and retaining these long-term dependencies, enabling them to handle such data types more effectively. In recent years, LSTM networks have also been applied to deep learning-based side-channel attacks, yielding certain achievements. By introducing gating mechanisms to selectively remember, forget, and output information, LSTM networks can more accurately learn and predict patterns and regularities within sequences when addressing sequence data problems. As illustrated in Fig 8, after the gating mechanism, the sigmoid activation function can constrain the values of the input gate *I*_*i*_, forget gate *F*_*i*_, and output gate *O*_*i*_ within the range (0,1), while the tanh activation function can constrain the values of candidate memory units and hidden states within the range (-1,1). If the forget gate *F*_*i*_ remains at 1 and the input gate remains at 0, the memory unit *C*_*i*−1_ from the previous time step will be gradually passed on to the current time step’s memory unit *C*_*i*_ as time progresses.

By controlling the transmission state through gating states, remembering what needs long-term retention, and forgetting unimportant information, LSTM networks are beneficial for many tasks requiring “long-term memory”. The gating mechanism greatly alleviates the vanishing gradient problem, simplifies parameter tuning complexity, and provides feature filtering, enriching the representation information of vectors. In our proposed optimization method, the LSTM network is further optimized and integrated as a submodule in the new model. The ablation experiment assesses the impact of the module on the model performance by removing the module step by step. The experimental results show that the presence of the LSTM module significantly improves the model’s performance in processing time-series data, especially in capturing complex dependency patterns in side-channel attacks. This further validates the importance and applicability of LSTM in the task of side-channel attack and proves its superiority in solving the long-term dependency problem.

The proposed model structure in this paper primarily consists of three components: an encoder, a decoder, and an output layer. The encoder section comprises convolutional layers, batch normalization (BN) layers, and LSTM layers. The convolutional layers are employed to extract local features from the input data. The primary role of BN layers is to expedite the convergence speed of the network. Meanwhile, LS-TM layers are utilized to capture the temporal dependencies in the time series data. As the network deepens, the dimensions of the features increase, and the spatial resolution decreases.

The decoder part restores the feature maps to their original size through convolutional layers and upsampling layers. Each layer of the decoder is concatenated with the corresponding output from the encoder, facilitating “skip connections” that help the network recover more refined spatial information.

**Fig 9:**
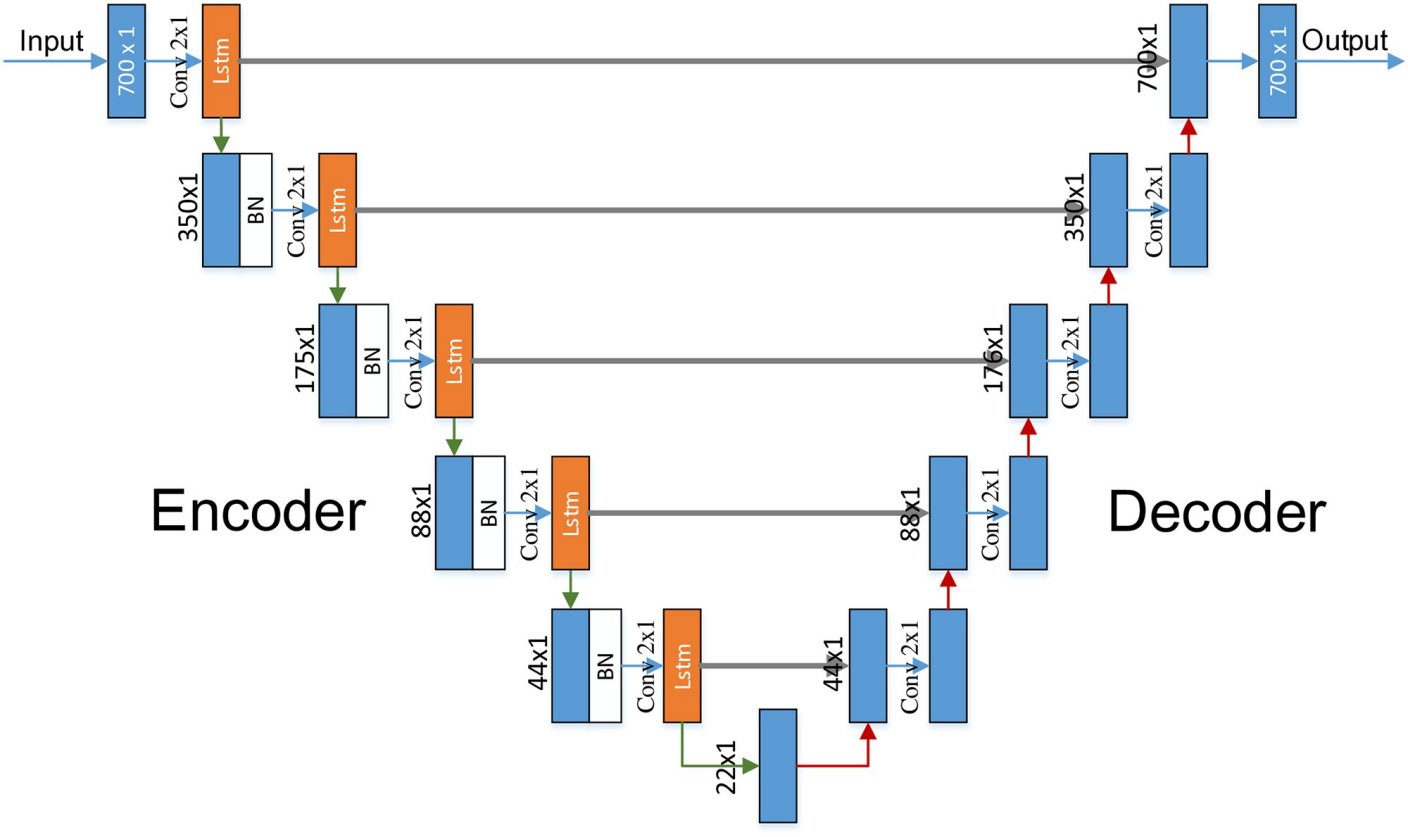
LU-Net model.

The output layer finally transforms the feature maps into output through convolutional layers. The overall structure is shown in Fig 9.

This model, when applied to the denoising of side-channel data, proves effective in capturing temporal dependencies and spatial details within time series or signal data. Given that side-channel data is a one-dimensional time series signal, applying this model to denoise side-channel data involves capturing the features of the input signal through the encoder, reconstructing the denoised signal thr-ough the decoder, and maintaining the spatial details and temporal coherence of the signal using skip connections and LSTM layers. Ultimately, the goal is to achieve denoising.

Due to the absence of noise, attackers can better identify and exploit information leaked by the physical device, leading to improved capabilities in obtaining encryption keys.

## Experiments and results

### Datasets

In side-channel attacks, common side-channel attack datasets are typically used for testing to assess the applicability of the designed network model. In this study, the proposed model was tested on the ASCAD dataset and the DPA-contest v4 dataset.

#### ASCAD dataset

The ASCAD dataset [4] provides power traces generated by shielding AES-128 implemented on the ATMega8515 microcontroller. In this study, we utilized the ASCAD.h5 dataset, extracted from the original power traces with complete synchronization.

It features first-order masking protection and includes 50,000 power traces for training and 10,000 power traces for attack, with each trace containing 700 features. Both the profiling and attack sets use the same fixed key.

#### DPA contest v4 dataset

DPA Contest v4 is the fourth edition of the DPA competition proposed by the Digital Electronics Systems Research Group at Télécom Paris, University of Paris-Saclay, in 2013. DPA Contest v4 is implemented by AES software with masking protection using a rotating S-box mask [23]. However, this scheme has first-order leakage, and when the value of the mask is known, attackers may consider this implementation unprotected and can directly recover the key [19]. Usually, sensitive variables are used:

$$\begin{array}{c} v^{\left(i\right)}(k^{*})=S-\text{box}[P_{0}^{i}⊕k^{*}]⊕M \end{array}$$ (4)

to identify each energy trace, where M denotes a known mask, $P_{0}^{i}$ denotes the first byte of the i-th plaintext, and k* is the key used. This dataset consists of 4,500 power traces for training and 500 power traces for attack, with each trace containing 4,000 features.

#### AES_RD dataset

The AES_RD dataset is a software implementation of the AES cryptographic algorithm using random delay guards and is a collection of electromagnetic energy traces captured using an electromagnetic probe when running on an 8-bit Atmel AVR microcontroller. The energy traces in this dataset are non-aligned energy traces, weakening the relationship between the energy traces and the leakage median and making attacks difficult. In this paper, the output of the first Sbox box of the cryptographic algorithm in the first round of encryption is chosen as the leakage median for the side channel attack on this dataset v, see Eq (5). This dataset has a total of 50,000 energy traces, out of which 40,000 traces are used as energy traces used in the modeling phase and the remaining 10,000 traces are used as energy traces used in the attacking phase and there are 3,500 sampling points for each energy trace.

$$\begin{array}{c} v=S-\text{box}(P_{1}⊕k) \end{array}$$ (5)

Where: is the input plaintext message of the first Sbox box and is the key used in the first round.

### Side-channel attacks

#### Attack results on the ASCAD dataset

**Fig 10:**
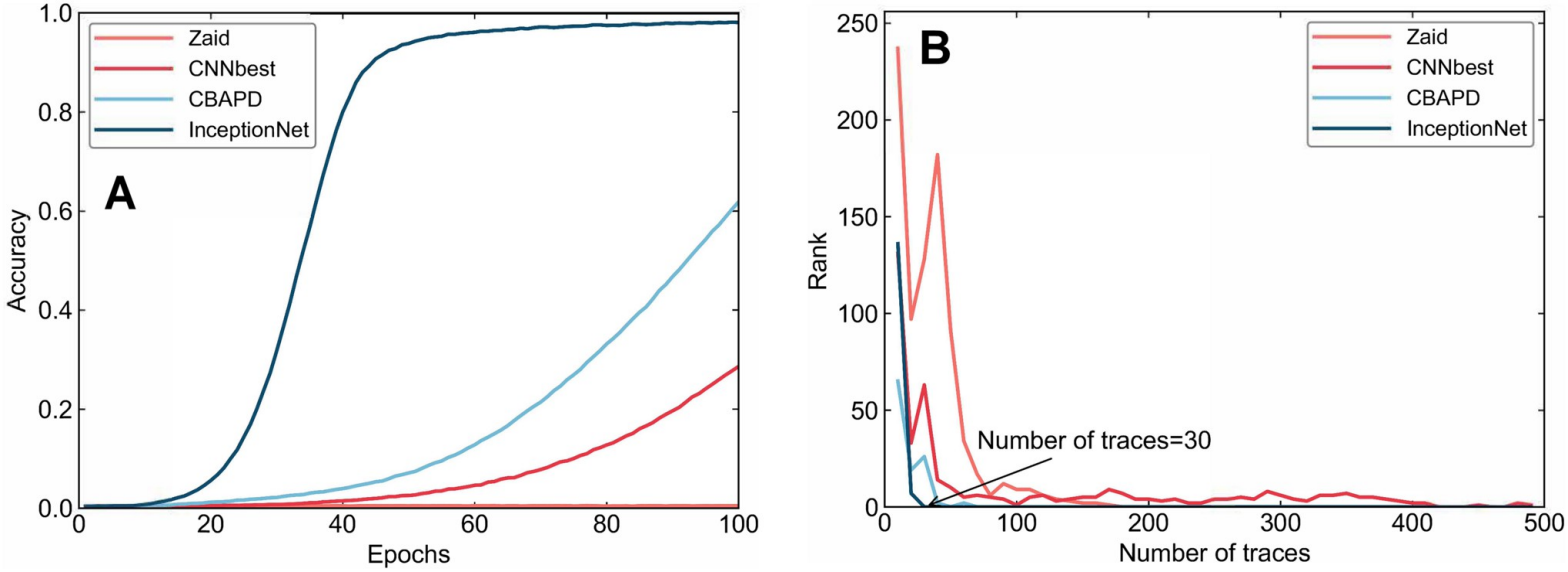
Comparison of attack results on the ASCAD dataset. A: Training Accuracy B: Attack Performance.

To validate the effectiveness of the proposed network in side-channel attacks, we conducted comparative experiments using several common deep learning models, namely Zaid, CNN_best, and CBAPD. Model training and side-channel attack efficiency were compared to analyze the side-channel attack performance of the models. The four network models were tested on both the ASCAD dataset and the DPA Contest v4 dataset. Training was performed for 100 epochs on the ASCAD dataset with a batch size set to 200, and attacks were executed under the same settings. The experimental results are shown in Fig 10.

From Fig 10A, it can be observed that, under the same number of training epochs, the training accuracy of the InceptionNet model is higher than that of the other three models. The comparison of attack efficiency is shown in Fig 10B. The network model proposed in [3] requires 170 energy traces for the rank to completely drop to 0 during the attack. The model proposed in [4] achieves a successful attack with 510 energy traces, while the model proposed in [5] needs 50 energy traces for a successful attack. In contrast, the model proposed in this paper only requires 30 energy traces to reduce the rank to 0. This indicates that the proposed network structure, capable of fusing multiscale features, exhibits high efficiency on the ASCAD dataset. A comparison of the specific parameters is shown in Table 2.

**Table 2:** Comparison of training parameters of ASCAD dataset.

Comparison Content	CNN_best [4]	Zaid [5]	CBAPD [6]	InceptionNet
accuracy	0.2858	0.0046	0.6179	0.9801
Activation function	ReLU	SeLU	ReLU	ReLU
Number of parameters	284,756,352	16,960	66,658,432	41,107,280
Number of traces	510	170	50	30
training time(s) /step	7	1	9	35

#### Attack results on the DPA contest v4 dataset

**Fig 11:**
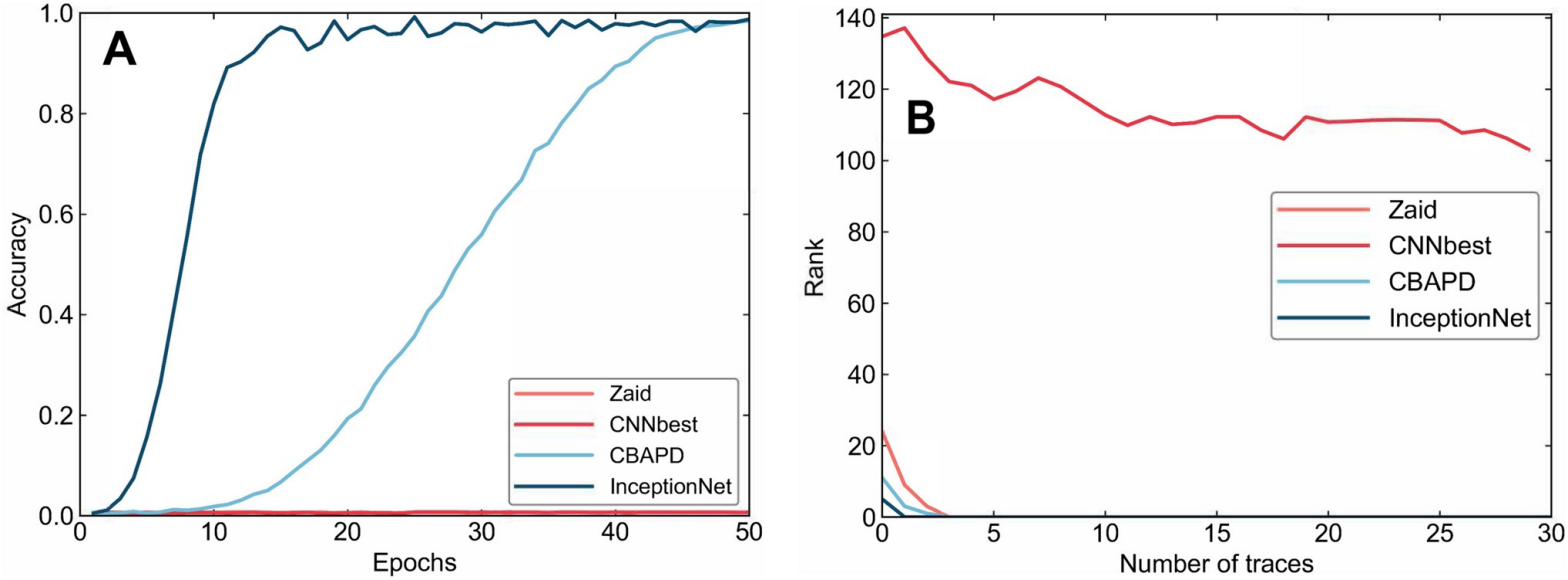
Comparison of attack results on the DPA contest v4 dataset. A: Training Accuracy B: Attack Performance.

Similar to the experiments on the ASCAD dataset, training was conducted for 50 epochs on the DPA contest v4 dataset with a Batch Size set to 200. Attacks were then executed under the same settings. The experimental results are shown in Fig 11.

From Fig 11A, while training with the InceptionNet model, it is evident that a remarkable accuracy of 0.98 is attained in just 18 epochs. In contrast, achieving similar accuracy with the CBAPD network necessitates 49 epochs. The comparison of attack performance is shown in Fig 11B, where InceptionNet achieves successful attacks with only 1 energy trace, while CBAPD and Zaid require 3 energy traces for a successful attack.

Table 3 lists the comparison of training parameters and attack effectiveness on the DPA Contest v4 dataset. Summarizing the results of the experiments, it can be concluded that the InceptionNet model proposed in this paper exhibits significantly higher efficiency and performance in side-channel attacks on the DPA contest v4 dataset compared to other common models based on convolutional neural networks.

**Table 3:** Comparison of training parameters of DPA contest v4 dataset.

Comparison Content	CNN_best [4]	Zaid [5]	CBAPD [6]	InceptionNet
accuracy	0.0071	0.0064	0.9842	0.9875
Activation function	ReLU	SeLU	ReLU	ReLU
Number of parameters	284,756,352	8,782	284,762,240	35,768,016
Number of traces	None	3	3	1
training time(s) /step	3	0.32	3.9	4.1

#### Attack results on the AES_RD dataset

**Fig 12:**
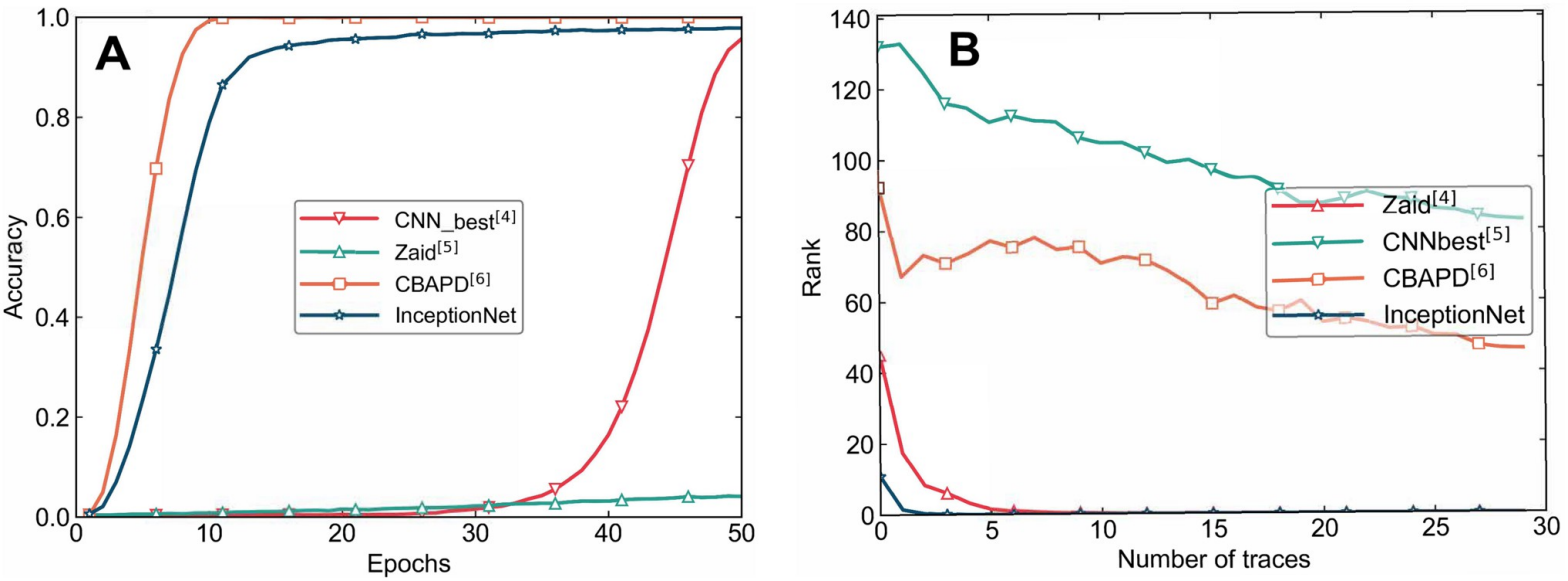
Comparison of attack results on the AES_RD dataset. A: Training Accuracy B: Attack Performance.

The experimental results comparing the Zaid, CNN_best, and CBAPD models with the InceptionNet model on the AES_RD dataset are shown in Fig 12A, and the specific data is detailed in Table 4. Additionally, the attack performance of these models is illustrated in Fig 12B.

**Table 4:** Comparison of training parameters of AES_RD dataset.

Comparison Content	CNN_best [4]	Zaid [5]	CBAPD [6]	InceptionNet
accuracy	0.0411	0.9566	1.0	0.9781
Activation function	ReLU	SeLU	ReLU	ReLU
Number of parameters	284,756,352	8,782	284,762,240	35,768,016
Number of traces	None	8	None	3
training time(s) /step	3	0.32	3.9	4.1

Based on the training results shown in Fig 12A, after 50 epochs, the Zaid model failed to converge. Although both the CNN_best and CBAPD models successfully converged, with the CBAPD model demonstrating a better convergence performance, both models failed to succeed in the attack due to overfitting. On the other hand, the InceptionNet model exhibited faster convergence, achieving an accuracy of 0.9781.

From Fig 12B and Table 4, it can be seen that for side-channel attacks using 30 power traces on the AES_RD dataset, both the CNN_best and CBAPD models failed to successfully extract the correct key. The Zaid model required 8 power traces to succeed, while the InceptionNet model only needed 3 power traces to complete the attack successfully. Compared to the other three models, the attack efficiency of the InceptionNet model significantly improved, achieving at least a 62.5% increase in efficiency.

Therefore, by analyzing the attack results of different models on the AES_RD dataset, it is evident that the InceptionNet model outperforms the other three models in terms of training and attack performance on the AES_RD dataset. Furthermore, the InceptionNet model demonstrates strong robustness against side-channel countermeasures in the AES_RD dataset, which includes random delay, further highlighting its capability to handle side-channel protection mechanisms effectively.

### Side-channel attacks with noisy

#### Gaussian noise

Gaussian noise stands out as the predominant type of noise present in side-channel power traces. Sour-ces of Gaussian noise can include transistors, data buses, transmission lines in oscilloscopes, and even the working environment. In terms of leakage information from power traces, the increase in noise level reduces the signal-to-noise ratio. Therefore, noise affects the effectiveness of attacks, meaning attackers need more power traces to obtain the device’s key information.

**Fig 13:**
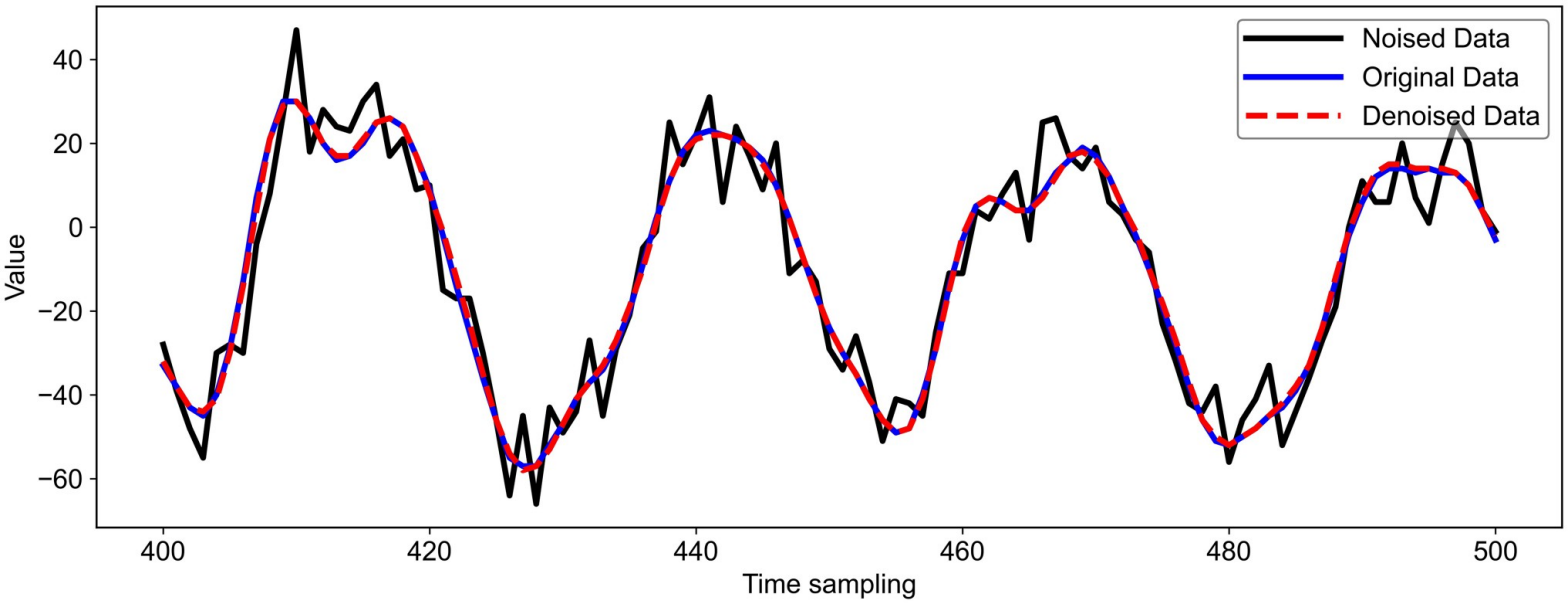
Using LU-Net model to remove gaussian noise.

**Fig 14:**
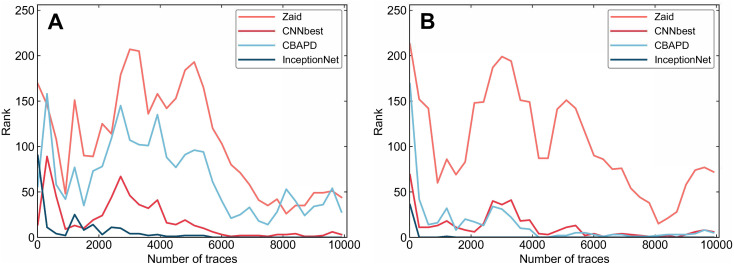
Side channel attack results. A: Denoised with DAE B: Denoised with LU-Net.

With the addition of Gaussian noise, the original energy trace is disturbed to some extent. Thus, the attack is much more difficult and even unsuccessful. After denoising the added noise dataset using LU-Net, as shown in Fig 13, a good fitting effect is presented between the denoised traces and the original energy traces. The denoising process effectively realizes a successful mitigation of noise in the original data. Subsequently, we train the model on the dataset after denoising using Denoising Auto-Encoder(DAE) denoising as well as the denoising model proposed in this paper, respectively, and then use the trained model to execute the attack, and the results of the model attack are shown in Fig 14.

The dataset is much more difficult to attack under the influence of noise, and the attack is even more unsuccessful. After we use DAE and LU-Net denoising, respectively, the attack effect is shown in Fig 14. As shown in Fig 14A, after using DAE denoising, the model proposed in this paper requires 5800 energy traces to reduce rank to 0. As shown in Fig 14B, after using LU-Net denoising, the model proposed in this paper requires only 1600 energy traces to reduce rank to 0. Moreover, the energy traces required by the CNN_best model and the CBAPD model to make Rank 0 are also reduced correspondingly, indicating that the LU-Net model proposed in this paper is more effective in attacking the data set with noise. is also reduced accordingly, which shows the efficiency of the LU-Net model proposed in this paper to remove Gaussian noise. The efficiency of side channel attacks is improved after removing Gaussian noise using the LU-Net model proposed in this paper.

#### Permutation noise

This paper employs the same method for implementing permutation noise as described in [15]: randomizing access to the S-box. By collecting trace segments associated with 16 S-box accesses and clustering them into 16 groups, a permutation effect is simulated. For the traces to be processed, a random group is chosen, and the attack trace portion (related to S-box processing) is replaced with a segment from that group.

**Fig 15:**
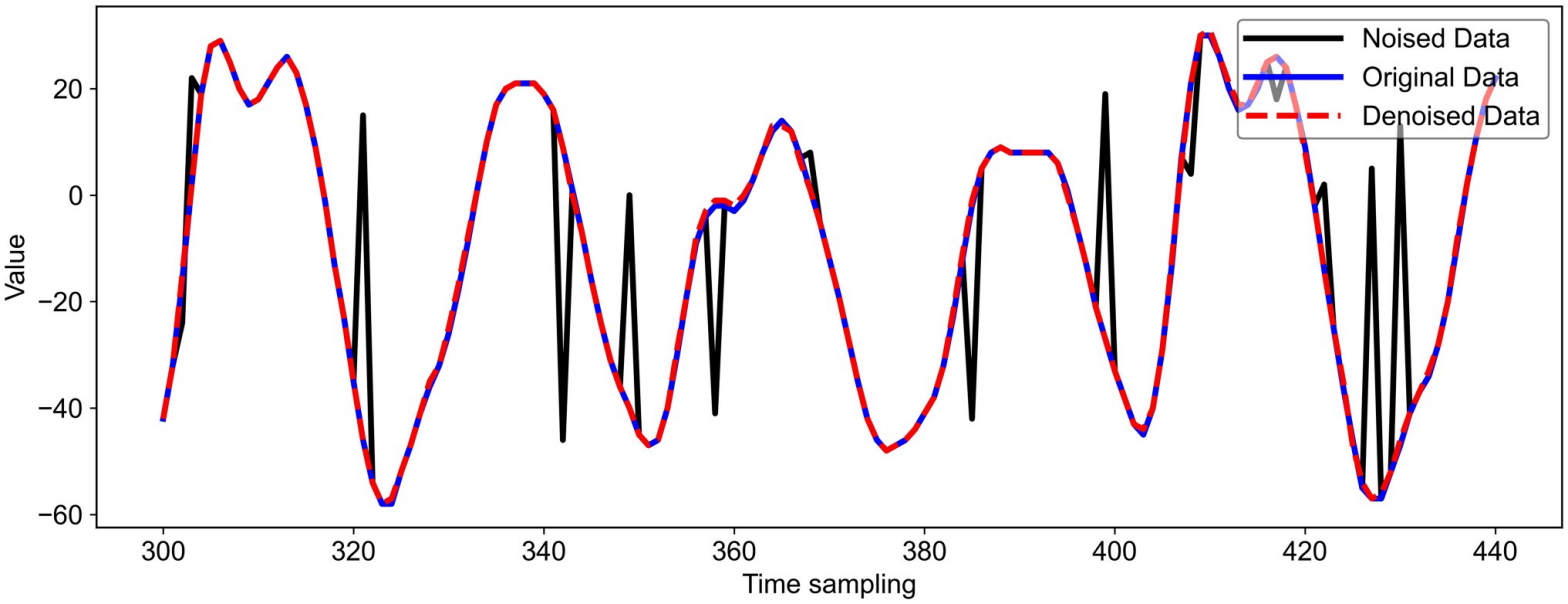
Using LU-Net model to remove permutation noise.

**Fig 16:**
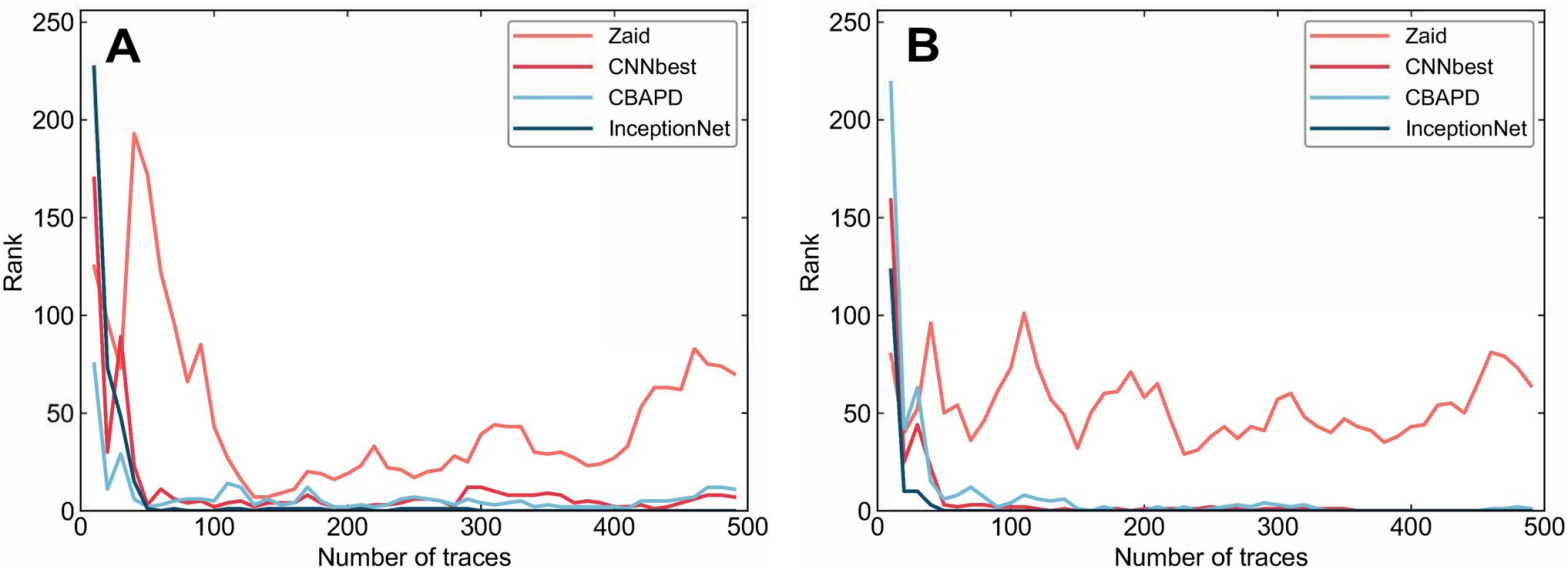
Side channel attack results. A: Denoised with DAE B: Denoised with LU-Net.

With this approach, it becomes more difficult for the attacker to select points of interest or locate intermediate data related to the S-box. The effect of adding disordered noise and denoising using the LU-Net model is shown in Fig 15. After denoising the noisy dataset using LU-Net, the denoised energy trace has an excellent fit to the original energy trace. Subsequently, the attack is carried out using the four attack models mentioned above, and the attack results are shown in Fig 16.

After adding the permutation noise, the attack difficulty of all four models is increased; after we use DAE and LU-Net denoising, respectively, the attack results are shown in Fig 16. As shown in Fig 16A, after using DAE denoising, the model proposed in this paper needs 290 traces to reduce rank to 0 completely, while as shown in Fig 16B, after using LU-Net denoising, the model proposed in this paper needs only 50 traces to reduce rank to 0. It is worth noting that the CNN_best model and the CBAPD model can reduce rank to 0 after using LU-Net denoising. The energy traces required to reduce rank to zero are also reduced accordingly, demonstrating the high efficiency of the LU-Net model proposed in this paper in removing disorganized noise.

#### Analysis of model adaptability in different noise environments

To evaluate the robustness of LU-Net in various noise environments, we conducted comparative experiments on the performance of the LU-Net model under Gaussian noise and shuffled noise. The experimental results are shown in Table 5.

**Table 5:** Comparison of denoising performance between LU-Net and DAE models.

*Denoising Model*	*Noise Type*	*Comparison* *Content*			
		*Parameters*	*Time*(*s*)/*step*	*MSE*	*Energy* *Traces*
DAE	Gaussian Noise	4,049,093	20	1.2657	5800
LU-Net	Gaussian Noise	4,256,321	24	1.1542	1600
DAE	Permutation Noise	4,049,093	20	1.7000	290
LU-Net	Permutation Noise	4,256,321	22	0.2271	50

As can be seen in Table 5, denoising with the LU-Net model results in a smaller mean square error (MSE), as well as a smaller number of energy traces required for a successful attack, than denoising with the DAE model. The relevance of Gaussian and disorganized noise is demonstrated by the fact that both significantly degrade the distinguishability of the signal, but LU-Net shows consistent advantages in handling both types of noise. For different noise conditions, future research could try multi-noise training to improve the model’s adaptability and robustness.

Through a series of experiments, we verified the effectiveness of the LU-Net model under two common side-channel noise conditions. Particularly, compared to traditional denoising models (such as DAE), LU-Net significantly improves the success rate of side-channel attacks with fewer traces. Additionally, LU-Net’s robustness in complex noise environments like Gaussian noise and shuffled noise further demonstrates its potential as an efficient denoising model. Compared to existing methods, LU-Net not only enhances denoising performance but also greatly reduces the computational cost of attacks, significantly improving attack efficiency.

## Conclusion

This paper systematically investigates the application of deep learning in side-channel attacks, emphasizing its potential as a high-performance data analysis method. Deep learning has demonstrated significant effectiveness in both the data preprocessing and attack phases, substantially enhancing the overall performance of side-channel attacks. In this study, we propose two novel deep learning-based network architectures specifically designed for denoising side-channel datasets and improving attack outcomes.

First, we introduce the InceptionNet model, which uses fewer training parameters compared to traditional CNNs. By processing input data in parallel, it achieves faster convergence and better representation of input data features. This approach addresses the challenges of high computational demand and inadequate data characterization often encountered in training side-channel attack models. Second, we introduce the LU-Net denoising model, which minimizes the detrimental effect of noise on attack efficiency, efficiently reconstructs denoised signals, and captures crucial input signal features.

To evaluate the performance of the proposed models, we conducted extensive experiments on the ASCAD and DPA Contest v4 datasets. The results confirm that the deep learning attack model proposed in this paper enhances attack efficiency by reducing the required number of traces, while the denoising model significantly mitigates the effect of noise, further improving attack performance.

## Supporting information

S1 Dataset (ZIP)

S1 File (DOCX)
