# Supplementary figures and images for: Deep learning-based improved side-channel attacks using data denoising and feature fusion
Source: PLoS One. 2025 Apr 9;20(4):e0315340. doi: 10.1371/journal.pone.0315340 (PMC11981128; doi:10.1371/journal.pone.0315340)

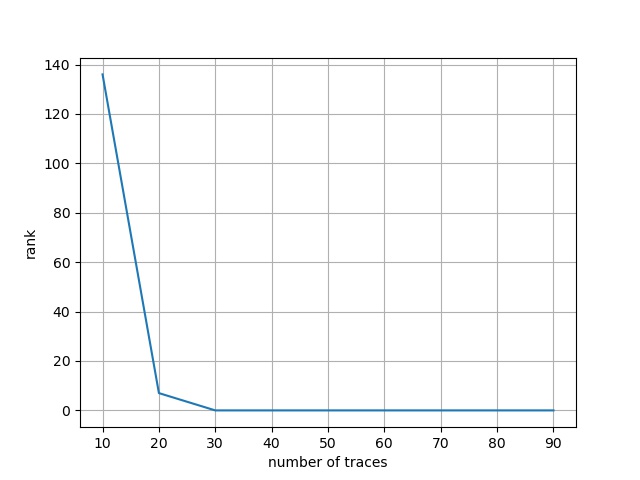

Supplement: S1 Dataset — (ZIP) [file pone.0315340.s001.zip › The minimal data set/ASCAD/fig/new.jpg]

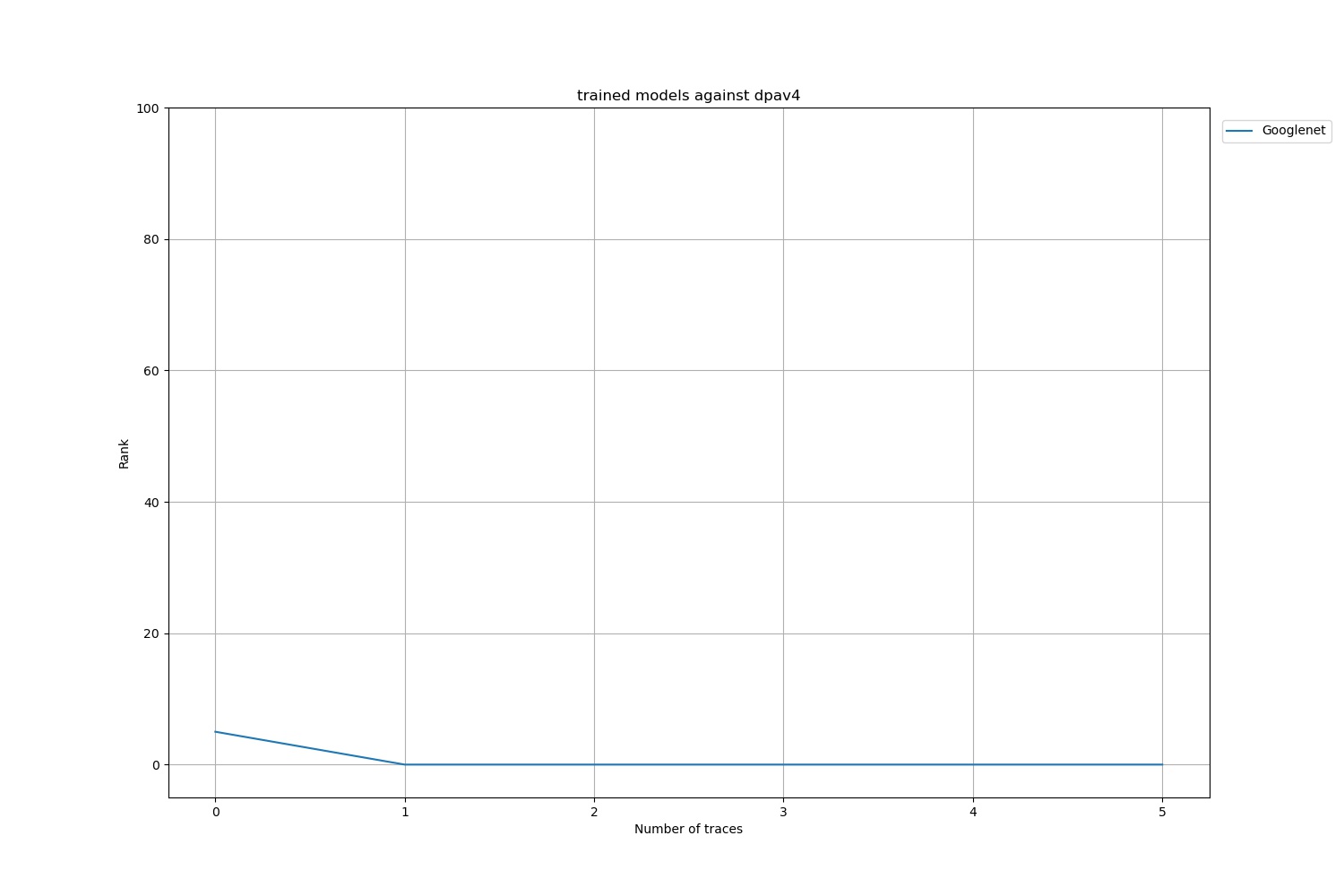

Supplement: S1 Dataset — (ZIP) [file pone.0315340.s001.zip › The minimal data set/DPA-contest v4/fig/1.jpg]
