# Supplementary material for: Deep learning-based improved side-channel attacks using data denoising and feature fusion
Source: PLoS One. 2025 Apr 9;20(4):e0315340. doi: 10.1371/journal.pone.0315340 (PMC11981128; doi:10.1371/journal.pone.0315340)
Supplement: S1 File — (DOCX) [file pone.0315340.s002.docx]

Supporting Information Description

Supporting information file name: The minimal data set.zip. It contains the code for the paper called Deep Learning-Based Improved Side-Channel Attacks Using Data Denoising and Feature Fusion. It consists of 4 parts.

1. ASCAD: This folder contains the code train_model.py and test_models.py for training the ASCAD dataset models, as well as the trained models in the ASCAD_trained_models folder for the readers to reproduce the results of this paper.
2. DPA-contest v4: This folder contains the code train_model.py and DPA_test_models.py for training the models of the ASCAD dataset, as well as the trained models in the DPAv4_trained_models folder for the readers to reproduce the results of this paper.
3. AES_RD: This folder contains the code train_model.py and test_models.py for training the models on the ASCAD dataset, as well as the trained models in the AES_RD_trained_models folder for the readers to reproduce the results of this paper.
4. Denoise: In this folder, we provide methods for adding Gaussian noise and shifting noise corresponding to Add_noise.py and methods for removing these two types of noise corresponding to LUnet_denoise.py. The denoised dataset using LUnet_denoise.py is then verified by using the same methods used to train the model for the ASCAD dataset. denoising effect of our method.

Additionally, the dataset used to train the model can be downloaded at the link below:

ASCAD datasets：https://github.com/ANSSI-FR/ASCAD

DPA-contest v4 datasets：http://www.dpacontest.org/v4/42_traces.php

AES_RD datasets：https://github.com/ikizhvatov/randomdelays-traces
